# Prevalence of Myofascial Trigger Points in Isolated Idiopathic Cervical Dystonia: A Possible Contributor to Pain, Movement and Disability

**DOI:** 10.1002/mdc3.14142

**Published:** 2024-06-19

**Authors:** Diego de‐la‐Hoz‐López, Víctor Gómez‐Mayordomo, María L. Cuadrado, Rocío García‐Ramos, Fernando Alonso‐Frech, José L. de‐la‐Hoz, César Fernández‐de‐las‐Peñas, Eva López‐Valdés

**Affiliations:** ^1^ Department of Medicine, School of Medicine Universidad Complutense Madrid Spain; ^2^ Department of Neurology, Hospital Clínico San Carlos Madrid Spain; ^3^ Department of Neurology, Institute of Neuroscience Hospital Universitario Vithas Madrid La Milagrosa, Vithas Hospital Group Madrid Spain; ^4^ Department of Odontology, School of Medicine Universidad CEU San Pablo Madrid Spain; ^5^ Department of Physical Therapy, Occupational Therapy, Physical Medicine and Rehabilitation Universidad Rey Juan Carlos Madrid Spain

**Keywords:** cervical dystonia, myofascial syndrome, pain, referred pain, trigger points

## Abstract

**Background:**

Myofascial trigger points (TrPs) are hypersensitive points located in a tight band of muscle that, when palpated, produce not only local pain but also referred (distant) pain. The role of TrPs in patients with cervical dystonia (CD) has not been investigated.

**Objective:**

To identify the presence of TrPs in patients with isolated idiopathic CD and their association with pain.

**Methods:**

Thirty‐one patients (74.2% women; age: 61.2 years, SD: 10.1 years) participated. TrPs were explored in the sternocleidomastoid, upper trapezius, splenius capitis, levator scapulae, anterior scalene, suboccipital, and infraspinatus muscles. Clinical features of CD were documented as well as the presence of pain. The severity of dystonia and its consequences were assessed using the Toronto Western Spasmodic Torticollis Rating Scale (TWSTRS).

**Results:**

The mean number of TrPs for each patient was 12 (SD:3), with no differences between patients with pain (n = 20) and those without pain (n = 11). Active TrPs were only found in patients with pain (mean: 7.5, SD:4). Latent TrPs were found in both groups but were more prevalent (*P* < 0.001) in patients without pain (mean: 11, SD:3.5) than in those with pain (mean: 5, SD:3.5). The number of active TrPs or latent TrPs was positively associated with the TWSTRS disability subscale and the TWSTRS total score. The number of active, but not latent, TrPs was associated with worse scores on the TWSTRS pain subscale.

**Conclusion:**

Active TrPs were present in patients with CD reporting pain, while latent TrPs were present in all CD patients, irrespective of their pain status. The numbers of active/latent TrPs were associated with disability. TrPs could act as pain generators in CD and also contribute to the involuntary muscle contractions characteristic of dystonia.

Dystonia is a clinical syndrome characterized by involuntary muscle contractions resulting in sustained or intermittent twisting movements or abnormal postures. Dystonic movements are typically patterned and can be initiated and/or worsened by voluntary actions, often associated with overflow muscle activation.^1^, ^2^ Dystonia is a heterogeneous disorder that can have different anatomic distributions and can be inherited (with a proven genetic origin), acquired (due to a known specific cause), or idiopathic.^2^

Isolated idiopathic cervical dystonia (CD) is a focal dystonia of unknown origin that involves the muscles of the neck. The movements may cause horizontal head rotation (torticollis), lateral deviation (laterocollis), head extension (retrocollis), head flexion (anterocollis), or a combination of directions.^3^ CD develops and worsens during the first months or years until the head deviation remains stable; however, spontaneous remissions can be observed in 10% of cases.^4^

Increasing attention is being paid to pain in dystonia as a relevant non‐motor manifestation of the disease. In fact, specific pain scales and classification systems are being developed for patients with dystonia.^5^, ^6^ Of all the focal forms of dystonia, CD is the most commonly associated with pain. Up to 89% of patients with CD may report pain, and more than 70% may rate their pain as moderate to severe.^7^ The pathophysiology of pain in CD remains largely unknown. Traditionally, pain has been attributed to sustained muscle contraction and the sudden twisting motion of the neck, which may cause tissue ischemia and local release of nociceptive substances. However, no correlation between CD related‐pain and the degree of muscle contraction has been found.^8^ Pain in CD is described as diffuse, sharp, shooting, or burning pain that is felt primarily in the neck and may extend to the shoulder, the upper back region, the side of the head deviation and the ipsilateral arm.^9^, ^10^ Therefore, it seems plausible that pain in CD could also be attributed to referred muscle pain linked to a coexisting myofascial syndrome.

Over the past few decades, there has been a growing recognition of myofascial pain as a distinct and important category within the field of muscle pain. Myofascial pain is featured by the presence of myofascial trigger points (TrPs), defined as hypersensitive spots located in a tense band of muscle whose palpation not only causes local pain but also referred (distant) pain.^11^ On examination, a local twitch response to palpation (ie, an involuntary brief, sudden contraction of the muscle taut band) is also characteristic. Clinically, TrPs are classified into active TrPs, which reproduce all or part of the patient's symptoms when referred pain is elicited, and latent TrPs, in which the referred pain does not replicate any symptom familiar/usual to the patient.^11^ Both active and latent TrPs provoke motor dysfunctions, including muscle weakness, inhibition, increased motor irritability, spasm, imbalance, and altered motor recruitment in the affected muscle or in functionally related muscles.^12^, ^13^ Thus, it is reasonable to hypothesize that patients with CD may have TrPs in the affected muscles, potentially contributing to both their pain and their postural changes. However, the presence of TrPs in patients with CD has not yet been investigated.

This study aimed to explore the presence of TrPs in patients with CD and its association with the presence of pain. We hypothesized that referred pain provoked by active TrPs could contribute to pain symptomatology experienced by these patients. In addition, we hypothesized that CD patients without pain might also have latent TrPs in the neck muscles that could contribute to dystonic cervical deviation.

## Methods

### Participants

A descriptive, cross‐sectional, observational, study was conducted. Patients with CD were consecutively recruited from the Movement Disorders Unit of the Neurology Department at Hospital Clínico San Carlos, Madrid, between November 2022 and October 2023. The inclusion criteria were: (1) Adults aged between 18 and 80 years; (2) Diagnosis of idiopathic isolated CD according to international consensus criteria^2^; (3), Mobile (non‐fixed) pattern of dystonia; (4) At least 12 weeks since the last infiltration with botulinum toxin (BT) for the treatment of dystonia; and (5) Written informed consent. The exclusion criteria were: (1) Diagnosis of secondary dystonia or clinical data suggesting a secondary etiology (such as the presence of structural lesions or examination findings consistent with functional dystonia); (2) Having received physical therapy treatment including acupuncture and/or dry needling in the last month; (3) Having received local injections within the last 3 months; (4) Modifications in pharmacological treatment for dystonia in the last month; (5) History of surgery, whiplash or cervical radiculopathy; (6) Other concomitant severe systemic disease; (7) Any other chronic pain syndrome; or (8) Inability to understand and/or complete the informed consent or to perform an adequate recording of the symptoms.

This study was approved by the Clinical Research Ethics Committee of Hospital Clínico San Carlos (code 22/255‐E). All participants were provided with written and verbal information about the study and signed an informed consent form.

### Demographic and Clinical Variables

The demographic and clinical variables collected included: age, sex, time of evolution of CD, head deviation, the presence or absence of head tremor, the presence or absence of pain, as well as the time receiving BT and the time elapsed since the last BT infiltration.

For patients who reported the presence of pain, the following data were recorded: the distribution of the pain, the side of the pain, the intensity of the pain in the previous week on a numerical pain rating scale (NPRS, 0: no pain, 10: the worst pain imaginable), and the temporal pattern of the pain (continuous or intermittent).

The severity of dystonia and its consequences (related‐disability and pain) were assessed using the Toronto Western Spasmodic Torticollis Rating Scale (TWSTRS). The total score of the TWSTRS ranges from 0 to 85 and is the sum of the scores of three subscales: severity of dystonia (0–35), disability (0–30) and pain (0–20). TWSTRS is the most widely used scale in CD and it has shown good interobserver reliability, convergent validity, and sensitivity to change.^14^

### Trigger Point (TrP) Examination

**Figure 1:**
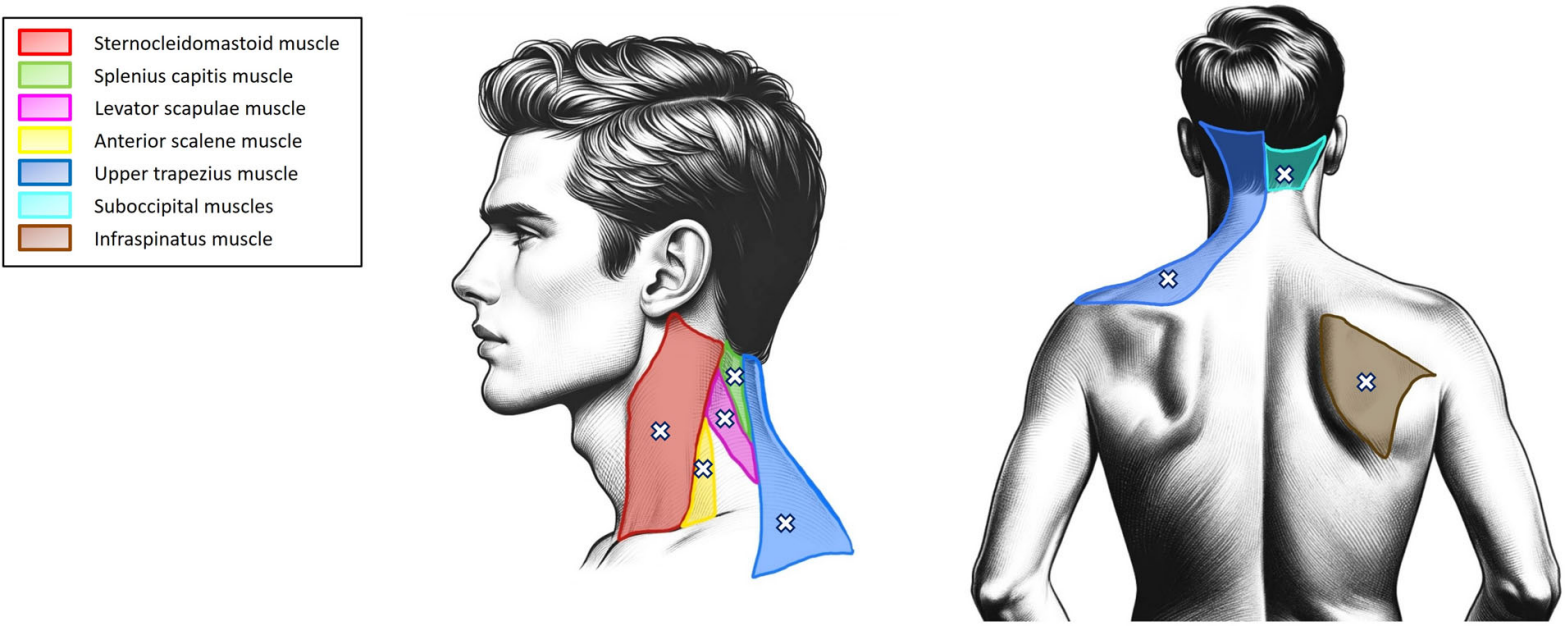
Muscles palpated for the presence of myofascial trigger points (TrPs) in this study. See text for more details on the procedure.

All patients were examined by the same researcher, after extensive training and proven experience in the evaluation of myofascial TrPs. The examiner was always blind to the patient's pain status at the time of the examination. On the day of the evaluation, the patient was not allowed to take any analgesics or muscle relaxants. The same muscles were bilaterally explored in all patients. Cervical and upper back muscles that could contribute to the pattern of pain experienced by CD patients (neck, shoulder, upper back, head and/or arm pain) were selected (Fig. 1).^8^, ^9^, ^10^, ^11^ To examine for the presence of TrPs, a systematic clinical evaluation was conducted to avoid continuous postural changes in the patient. Thus, the sternocleidomastoid, upper trapezius, splenius capitis, anterior scalene and suboccipital muscles were explored with the patient lying supine, the levator scapulae muscle was evaluated with the patient lying in lateral decubitus, whereas the infraspinatus muscle was explored with the patient in a prone position. The diagnosis was performed according to the following diagnostic criteria: presence of a hypersensitive spot within a taut band of the muscle and referred pain sensation elicited by manual stimulation of the spot.^15^ Pressure was applied with a specific technique for each muscle studied: for the sternocleidomastoid and trapezius muscles, the examiner used his thumb and index finger as a clamp; for the splenius, anterior scalene, suboccipital, levator scapulae and infraspinatus muscles, he used his index finger, and for the suboccipital muscles, he applied pressure with the second to fourth fingers of his hand. A TrP was considered active when the evoked referred pain during examination reproduced any symptom experienced by the patient and the pain was recognized as a familiar sensation.^13^ A TrP was classified as latent when the evoked referred pain did not reproduce any symptom experienced by the patient and the pain was not recognized as a familiar sensation.^15^

### Statistical Analysis

Mean and standard deviation (SD) are presented for quantitative data, whereas absolute values and percentages are presented for categorical variables. The chi square (*X* ^2^) test was used to analyze differences in the distribution of TrPs (active or latent) for each muscle between CD patients with and without pain. Similarly, the chi square (*X* ^2^) test was used to analyze the differences in the distribution of TrPs (active or latent) for each muscle among patients with CD according to the pattern of head deviation. Differences in the number of TrPs (active or latent) between patients with and without pain were assessed with the unpaired Student *t*‐test. The unpaired Student's *t*‐test was also used to compare the presence of TrPs between patients with and without head tremor. Finally, the Pearson correlation test (*r*) was used to determine the association between the number of TrPs and clinical variables relating to CD. Correlations were considered weak when r was <0.3, moderate when 0.3< *r* < 0.7, and strong when *r* > 0.7. The statistical analysis was conducted at a 95% confidence level, with a *P*‐value <0.05 being considered statistically significant.

## Results

Forty‐six patients attending the Movement Disorders Unit of the Neurology Department of Hospital Clínico San Carlos, Madrid, were screened for eligibility criteria. Fifteen patients (32.6%) were excluded for the following reasons: four had segmental dystonia with craniofacial or upper limb musculature involvement, three met criteria for functional dystonia, one had tardive cervical dystonia, one had cervical radiculopathy, and six refused to participate. Finally, 31 patients (74.2% women; mean age: 61.2 years, SD: 10.1 years) were included. In our sample, 20 patients reported pain (prevalence of pain: 64.5%); of these, 20 patients (100%) felt pain in the neck, 11 (55%) in the upper back, 10 (50%) in the head, 10 (50%) in the shoulder, and 5 (25%) in the arm. Table 1 summarizes the demographic and clinical features of the sample.

**TABLE 1:** Demographic and clinical features of the sample (n = 31)

**Demographic variables**	
Age (years), mean (SD)	61.2 (10.1)
Sex, n (%)	
Female	23 (74.2)
Male	8 (25.8)
**Clinical variables**	
Time since onset of CD (years), mean (SD)	15.4 (12.1)
Time since start of BT treatment (years), mean (SD)^a^	11.1 (9.5)
Head deviation, n (%)	
Torticollis	14 (45.2)
Laterocollis	10 (32.3)
Anterocollis	5 (16.1)
Retrocollis	2 (6.5)
Head tremor, n (%)	23 (74.2)
Patients with pain, n (%)	20/31 (64.5)
Neck pain	20/20 (100)
Upper back pain	11/20 (55)
Head pain	10/20 (50)
Shoulder pain	10/20 (50)
Arm pain	5/20 (25)
Side of pain, n (%)	
Right	9/20 (45)
Left	6/20 (30)
Both	5/20 (25)
Intensity of pain (NPRS, 0–10), mean (SD)	6 (1.8)
Temporal pattern of pain, n (%)	
Continuous	11/20 (55)
Intermittent	9/20 (45)
TWSTRS	
Severity score, mean (SD)	13.5 (5.1)
Disability score, mean (SD)	6.4 (6.3)
Pain score, mean (SD)	8 (6.9)
Total score, mean (SD)	27.9 (13.4)

Abbreviations: BT, botulinum toxin; CD, cervical dystonia; NPRS, Numerical Pain Rate Scale (0: no pain, 10: the worst pain imaginable); SD, standard deviation; TWSTRS, Toronto Western Spasmodic Torticollis Rating Scale.

^a^ 17 patients were assessed at 3 months and 14 patients at 4 months after their last BT injection.

In all participants, the examination showed the presence of myofascial TrPs in several of the muscles tested. Overall, the mean number of TrPs for each patient was 12 (SD: 3), of which 5 (SD: 4.5) were active TrPs and the remaining 7 (SD: 4.5) were latent TrPs. Active TrPs were found in all patients with pain (mean: 7.5, SD: 4) and in none of the patients without pain. Latent TrPs were detected in both pain and non‐pain patients, although they were significantly more prevalent (*P* < 0.001) in patients without pain (mean: 11, SD: 3.5) compared to those experiencing pain (mean: 5, SD: 3.5). There were no significant differences (*P* = 0.154) in the total number of TrPs between patients with pain (mean: 12.5, SD: 2.5) and those without pain (mean: 11, SD: 3.5). No differences were found in the number of TrPs (total, active or latent) between patients with head tremor (mean total: 11.8, SD: 3.1) and those without head tremor (mean total: 11.9, SD: 2.5; *P* = 0.968).

The number of active TrPs was positively associated with TWSTRS disability (*r* = 0.637, *P* < 0.001) and TWSTRS pain (*r* = 0.770, *P* < 0.001) subscales as well as the TWSTRS total score (*r* = 0.664, *P* < 0.001), but not with the TWSTRS severity subscale (*r* = 0.089, *P* = 0.685). The number of latent TrPs was also significantly and positively associated with the TWSTRS disability subscale (*r* = 0.629, *P* < 0.001) and the TWSTRS total score (*r* = 0.509, *P* < 0.001), but not with the TWSTRS severity subscale (*r* = 0.268, *P* = 0.145) or the pain subscale (*r* = 0.139, *P* = 0.559). In summary, a greater number of active or latent TrPs was associated with worse scores on the TWSTRS disability subscale and the TWSTRS total score. On the other hand, a higher number of active, but not latent, TrPs was associated with a worse score on the TWSTRS pain subscale.

Table 2 summarizes the distribution of TrPs in patients with CD with and without pain. All the examined muscles exhibited active or latent TrPs in the same proportion without differences between them. Likewise, no differences in the distribution of TrPs were found according to the deviation pattern (data not shown), probably due to the small number of subjects in some comparisons (all, *P* > 0.214).

**TABLE 2:** Number and percentage of patients with active and latent myofascial TrPs in each of the muscles explored

Muscles	Patients with pain (n = 20)		Patients without pain (n = 11)	
	Right side	Left side	Right side	Left side
Sternocleidomastoid				
Active TrPs, n (%)	13 (65)	12 (60)	0 (0)	0 (0)
Latent TrPs, n (%)	6 (30)	7 (35)	10 (91)	9 (82)
No TrPs, n (%)	1 (5)	1 (5)	1 (9)	2 (18)
Upper trapezius				
Active TrPs, n (%)	14 (60)	12 (60)	0 (0)	0 (0)
Latent TrPs, n (%)	6 (30)	6 (30)	11 (100)	10 (91)
No TrPs, n (%)	0 (0)	2 (10)	0 (0)	1 (9)
Splenius capitis				
Active TrPs, n (%)	11 (55)	10 (50)	0 (0)	0 (0)
Latent TrPs, n (%)	7 (35)	7 (35)	8 (73)	10 (91)
No TrPs, n (%)	2 (10)	3 (15)	3 (27)	1 (9)
Levator scapulae				
Active TrPs, n (%)	14 (70)	10 (50)	0 (0)	0 (0)
Latent TrPs, n (%)	5 (25)	7 (35)	10 (91)	9 (82)
No TrPs, n (%)	1 (5)	3 (15)	1 (9)	2 (18)
Anterior scalene				
Active TrPs, n (%)	7 (35)	7 (35)	0 (0)	0 (0)
Latent TrPs, n (%)	12 (60)	10 (50)	7 (64)	8 (73)
No TrPs, n (%)	1 (5)	3 (15)	4 (36)	3 (27)
Suboccipital				
Active TrPs, n (%)	12 (60)	9 (45)	0 (0)	0 (0)
Latent TrPs, n (%)	5 (25)	5 (25)	7 (64)	6 (54.5)
No TrPs, n (%)	3 (15)	6 (30)	4 (36)	5 (45.5)
Infraspinatus				
Active TrPs, n (%)	11 (55)	7 (35)	0 (0)	0 (0)
Latent TrPs, n (%)	6 (30)	10 (50)	7 (64)	8 (73)
No TrPs, n (%)	3 (15)	3 (15)	4 (36)	3 (27)

## Discussion

Previous studies have documented the presence of myofascial TrPs in patients with musculoskeletal pain conditions,^16^ as well as in patients with neurological disorders, particularly stroke.^17^ In fact, the presence of TrPs in individuals with chronic cervical pain such as whiplash‐associated disorders or mechanical neck pain is consistent in the literature.^18^ However, this is the first study to analyze the presence of TrPs in patients with idiopathic CD. In our sample, all the patients reporting pain exhibited active TrPs. In addition, latent TrPs were found in the entire sample, both in patients with pain and in those without pain. Moreover, the number of latent or active TrPs was significantly associated with dystonia‐related disability.

In our study, we observed that patients with CD, remarkably those without pain, had latent TrPs across a diverse range of muscles involved in dystonic movements. These results support the idea that TrPs could play a role in the pathophysiology of some of the motor disturbances characterizing dystonia. Indeed, current evidence indicates that TrPs can disrupt modular motor control.^12^ Latent trigger points are associated with increased intramuscular electromyographic activity in both synergistic and antagonistic muscles,^19^, ^20^ suggesting altered muscle activation. In fact, the etiology of TrPs has been linked to a motor endplate dysfunction, causing sustained depolarization of the postsynaptic membrane and subsequent shortening of sarcomeres in proximity to the motor endplate.^21^ A new hypothesis has been proposed to explain the development of TrPs, based on the concept that pre‐existing myoprotective feedback mechanisms fail to protect the musculature under certain conditions, such as intense muscle activity.^22^ Therefore, the presence of TrPs could be facilitated by, and also contribute to, the involuntary muscle contractions seen in patients with CD.

Three major neurophysiological systems have been described in dystonia pathogenesis: a loss of inhibition at a multi‐segmentary level (spinal cord, brainstem, cortex), abnormalities in sensorimotor integration and maladaptive plasticity.^23^ Also, it is known that dysfunction in muscle spindle afferents and abnormal sensory inputs can modify or reinforce dystonic movements.^24^ The relationship of muscle spindle, TrPs and myofascial pain is well known.^25^ Thus, the presence of muscle TrPs might act in closed‐loop sensory feedback reinforcing dystonic central patterns. Accordingly, it is possible that TrPs, albeit latent, can contribute to the dystonic movements seen in CD. This hypothesis is further supported by the fact that the number of latent TrPs was associated with higher CD‐related disability. However, we did not find an association between the increased presence of TrPs and the severity of motor manifestations of dystonia or the pattern of cervical deviation, maybe due to sample size limitations. These issues need to be addressed in future studies.

This exploratory study also supports a role of active TrPs in CD‐related pain. In fact, active TrPs were detected in all the patients with pain, and in none of the patients without pain. TrPs were classified as active when the referred pain induced by palpation reproduced the pain patterns experienced by CD patients. Therefore, TrPs seem to be a pain generator in patients with CD. The presence of pain in CD is thought to be caused by both peripheral and central mechanisms.^26^ TrPs may contribute to pain through several peripheral mechanisms, including sustained muscle contraction, local ischemia, and the local release of inflammatory and other algogenic substances.^27^ In addition, active TrPs may contribute to central mechanisms, as they may promote central sensitization, ie, altered nociceptive processing.^28^ In such a scenario, the presence of nociception from active TrPs could excite spinal and supra‐spinal structures resulting in altered pain processing. Other pathophysiological mechanisms already described in dystonia may also contribute to abnormal inhibitory pain modulation.^29^

The presence of TrPs in patients with CD may have several implications for clinical practice. Botulinum toxin (BT) infiltrations are the treatment of choice for CD^30^ and are also widely used as treatment for TrP associated pain.^31^ The effect of BT is based on inhibiting the release of acetylcholine (ACh) in the motor endplate and the release of pain‐related neuropeptides from the sensory nerve endings.^32^ It would be conceivable that improvements seen in patients with CD after BT injection (particularly in pain) might be partially related to an improvement in myofascial syndrome. BT is normally injected into the musculature involved in the abnormal posture, adapting the number of muscles injected and the number of injection sites to the clinical situation of each patient with CD; specific BT injections directed to active o latent TrPs might have an additional effect in pain or postural abnormalities in dystonia. In addition, the use of specific therapies for TrPs, such as dry needling, local anesthetic injections, and manual therapy or other non‐invasive therapeutic interventions^33^, ^34^, ^35^ could serve as adjuvant therapy in patients with CD, maybe allowing for a reduction in BT doses or enhancing its effect between infiltrations. However, no conclusions can be drawn from this study regarding the use of these therapies in CD, whose efficacy should be evaluated in future clinical trials.

This is the first study investigating TrPs in CD, but potential limitations should be recognized. First, the sample is limited to just one hospital and could be considered relatively small. New studies including large samples are needed to confirm or refute our results. Second, the heterogeneity of the sample did not allow any comparison between different CD deviation patterns. Third, all participants were patients regularly treated with BT injections, which can potentially affect the presence of TrPs. Nevertheless, data collection was carried out at least 3 months after the last BT injection and, in any case, the presence of TrPs was clear in our sample. Finally, a limitation of this study is the lack of non‐CD controls. Pain‐free controls would not have active TrPs, but it would be interesting to compare the prevalence of latent TrPs between CD patients and healthy subjects. Furthermore, to clarify whether TrPs may play a differential role in the pathophysiology of CD, it would be useful to compare the prevalence of both active and latent TrPs between patients with CD and patients with other painful neck disorders to try to identify a dysfunctional pattern.

In conclusion, this study revealed that active TrPs were present in patients with CD reporting pain, while latent TrPs were found in all patients with CD, irrespective of their pain status. The numbers of active and latent TrPs were significantly associated with CD‐related disability. Myofascial TrPs could act as pain generators in CD and could also contribute to the involuntary muscle contractions characteristic of dystonia. We hypothesize that targeting TrPs would be an effective therapeutic approach for patients with CD.

## Author Roles

(1) Research project: A. Conception, B. Organization, C. Execution; (2) Statistical Analysis: A. Design, B. Execution, C. Review and Critique; (3) Manuscript: A. Writing of the first draft, B. Review and Critique.

D.H.L.: 1A, 1B, 1C, 2B, 2C, 3A, 3B

V.G.M.: 1A, 1B, 1C, 2C, 3A, 3B

M.L.C.: 1A, 1B, 2A, 2C, 3A, 3B

R.G.R.:1B, 1C, 3A, 3B

F.A.F.: 1B, 1C, 3A, 3B

J.L.H.: 2C, 3A, 3B

C.F.P.: 1A, 1B, 2A, 2B, 2C, 3A, 3B

E.L.V.: 1A, 1B, 2A, 2C, 3A, 3B

## Disclosures

**Ethical Compliance Statement:** This study was approved by the Clinical Research Ethics Committee of Hospital Clínico San Carlos (code 22/255‐E). All participants provided written informed consent form. We confirm that we have read the Journal's position on issues involved in ethical publication and affirm that this work is consistent with those guidelines.

**Funding Sources and Conflict of Interest:** The authors declare that there are no funding sources or conflicts of interest relevant to this work.

**Financial Disclosures for the previous 12 months:** The authors declare that there are no additional disclosures to report.
